# Pharmacokinetics and Tissue Distribution of Enrofloxacin and Its Metabolite Ciprofloxacin After a Single Intraperitoneal Administration in Black Rockfish (*Sebastes schlegelii*) at Two Water Temperatures

**DOI:** 10.3390/ani16193034

**Published:** 2026-09-26

**Authors:** Eun Ha Jeong, Jun Sung Bae

**Affiliations:** 1 Department of Aquatic Life Medicine, Kunsan National University, Gunsan 54150, Republic of Korea; dmsgk1532@kunsan.ac.kr; 2 Department of Aquatic Life Medicine, College of Marine Science, Gyeongsang National University, Tongyeong 53064, Republic of Korea

**Keywords:** enrofloxacin, pharmacokinetics, black rockfish, temperature

## Abstract

Enrofloxacin (ENR) is a fluoroquinolone antimicrobial used to treat bacterial diseases in aquaculture and is metabolized into ciprofloxacin (CIP). This study investigated the serum pharmacokinetics and tissue distribution of ENR and CIP in black rockfish (*Sebastes schlegelii*) following a single intraperitoneal administration of ENR 10 mg/kg at 13 °C and 22 °C. Absorption, distribution, and elimination of ENR were accelerated at 22 °C compared with 13 °C, with the elimination half–life decreasing from 35.75 h at 13 °C to 21.11 h at 22 °C. Kidney levels indicated higher ENR values than serum, liver, and muscle levels at both temperatures. CIP was formed only at low levels (2.13–8.05%), with greater formation at 22 °C. The temperature–elimination relationship observed after intraperitoneal administration was opposite to that reported for oral administration under the same conditions, suggesting the occurrence of flip–flop kinetics at high water temperature following oral administration. These findings indicate that both water temperature and route of administration should be considered when evaluating the pharmacokinetics of ENR in fish.

## Simple Summary

Changes in water temperature can affect how drugs behave in the body. We gave black rockfish an injection of enrofloxacin into the body cavity under two water temperature conditions, cold and warm, and measured how the drug and its breakdown product changed over time. We found that at the warmer temperature, the drug was absorbed and removed from the body more quickly, and among the tissues examined, the kidney showed the highest drug concentration. In addition, these results were the opposite of those from an earlier study that used oral administration instead of injection. These findings show that both water temperature and the method of drug administration need to be considered together when deciding how to treat bacterial infections in farmed fish, which can help fish farmers use antibiotics more safely and effectively.

## 1. Introduction

Enrofloxacin (ENR) is a synthetic fluoroquinolone antimicrobial agent developed for veterinary use, and is known to exhibit pharmacokinetic properties such as good oral absorption and wide tissue distribution, undergoing biotransformation during metabolism into ciprofloxacin (CIP), another fluoroquinolone antimicrobial [1,2,3]. ENR exhibits broad-spectrum antimicrobial activity against Gram-negative bacteria, Gram-positive bacteria, and *Mycoplasma* spp. [4,5], and owing to these properties, it is used in aquaculture to treat various bacterial diseases including those caused by *Aeromonas salmonicida*, *A*. *hydrophila*, *Renibacterium salmoninarum*, *Vibrio alginolyticus*, and *Streptococcus iniae* [6,7,8,9,10]. ENR is generally administered orally via feed at doses of 10–20 mg/kg body weight, and its pharmacokinetic properties have been evaluated under these dosing conditions in various fish species, including yellow croaker, crucian carp, tilapia, snakehead fish, largemouth bass, and brown trout [9,11,12,13,14,15]. These studies indicate that the pharmacokinetic properties of ENR can be influenced by various factors, including not only fish species but also water temperature and route of administration.

Black rockfish (*Sebastes schlegelii*) inhabits the coastal waters of the North Pacific and is a commercially important aquaculture species in Korea, China, and Japan [16,17,18]. Black rockfish is a species that prefers relatively low water temperatures; although it can survive temperatures up to 28 °C, it exhibits thermal tolerance within a range of 3–20 °C [17]. However, during aquaculture in Korea, this species is exposed to a wide range of water temperatures (7.8–26.7 °C) depending on seasonal variation [19]. Such wide-ranging temperature exposure acts as a physiological stressor for black rockfish and may induce changes in growth, metabolism, and immune function [20,21,22,23,24,25]. Therefore, to ensure the appropriate use of antimicrobial agents in black rockfish aquaculture, it is necessary to understand the pharmacokinetic properties of drugs in relation to changes in water temperature.

A substantial portion of an orally administered drug may be lost during transit through the gastrointestinal tract. In addition, drug absorption can be influenced by various factors, such as gastrointestinal transit time, pH, and the characteristics of gastrointestinal fluids, thereby contributing to variability in pharmacokinetics [26,27]. Intravenous administration, in contrast, involves no absorption process, as the drug is injected directly into the bloodstream, thereby allowing an accurate assessment of drug disposition unaffected by absorption [28]. However, in fish, intravenous administration can be technically challenging owing to anatomical characteristics such as vessel size and accessibility, with body size in particular being an important consideration [29]. Black rockfish are relatively small-bodied fish, typically cultured at body weights of 500 g or less, and to the best of our knowledge, no pharmacokinetic study using intravenous administration has been reported in this species. Intraperitoneal administration is classified as a parenteral route; however, the absorption rate of substances administered intraperitoneally is generally slower than that following intravenous injection. This is because drugs absorbed from the peritoneal cavity are primarily taken up by the mesenteric vessels, which drain into the portal vein and pass through the liver before reaching the systemic circulation [28]. Nevertheless, intraperitoneal administration may serve as a practical alternative route for evaluating the systemic pharmacokinetic properties of drugs in small fish species for which intravenous access is technically challenging [30].

In a previous study, we reported the pharmacokinetic properties of ENR in black rockfish under two representative water temperatures (13 °C and 22 °C), reflecting the seasonal average water temperatures along the western and southern coasts of Korea during winter-spring and summer-autumn, respectively [31]. Given the thermal tolerance range of black rockfish (3–20 °C) [17], 13 °C represents a near-optimal condition, whereas 22 °C exceeds this range and may impose thermal stress on the species. However, that study evaluated only the serum pharmacokinetics of ENR following oral administration, and thus could not distinguish whether the temperature-dependent pharmacokinetic patterns observed were attributable to genuine changes in systemic drug disposition or to variability in gastrointestinal absorption, since oral administration inherently combines both processes. This distinction is critical, as absorption-related variability, rather than true systemic disposition, may otherwise be misinterpreted as a temperature-dependent change in drug elimination. Because intravenous administration, which would allow this distinction by excluding the absorption process entirely, remains technically infeasible in small-bodied species such as black rockfish, intraperitoneal administration was adopted in the present study as a practical alternative that minimizes the influence of gastrointestinal absorption and provides a more direct assessment of the systemic pharmacokinetic properties of ENR. Therefore, in the present study, we applied the same dose (10 mg/kg body weight) and water temperature conditions (13 °C and 22 °C) to evaluate the temperature-dependent pharmacokinetic properties of ENR and its metabolite CIP following intraperitoneal administration, by analyzing concentration changes in serum and major tissues (liver, kidney, and muscle), and to compare these findings with those of the previous oral administration study. To our knowledge, this is the first study to characterize the tissue distribution and temperature-dependent pharmacokinetics of ENR and its metabolite CIP in black rockfish following intraperitoneal administration, thereby providing critical data to distinguish absorption-related variability from true systemic disposition and offering insight into how water temperature may influence the pharmacokinetic behavior of ENR and its metabolite in aquaculture settings.

## 2. Materials and Methods

### 2.1. Chemicals

ENR and CIP analytical standards were purchased from Sigma-Aldrich (St. Louis, MO, USA). HPLC-grade water, methanol, acetonitrile, and n-hexane were purchased from Merck (Rahway, NJ, USA). Sterile physiological saline and tricaine methanesulfonate (MS-222) were purchased from Sigma-Aldrich (St. Louis, MO, USA). For intraperitoneal administration, a commercially available veterinary medicinal product approved for use in aquaculture, Enpro 20 Solution (20% enrofloxacin; Samyang Anipham Co., Ltd., Seoul, Republic of Korea), was diluted with sterile physiological saline and used as the ENR dosing solution.

### 2.2. Animals and Experimental Design

A total of 156 Black rockfish (mean body weight: 54.4 ± 5.0 g) were purchased from a commercial aquaculture farm located in Boryeong, Chungcheongnam-do, Republic of Korea, and randomly distributed into two 600 L tanks (W 1.2 m × D 0.8 m × H 0.8 m), with 78 fish in each tank. Water temperature was controlled using an automatic temperature control system (DOV-882, DAEIL, Busan, Republic of Korea) and maintained at 13 ± 0.5 °C and 22 ± 0.5 °C in the respective tanks. During the experimental period, water quality parameters were monitored using a multiparameter meter (YSI 556, YSI Inc., Yellow Springs, OH, USA), and maintained at pH 7.0–8.0, salinity of 30 ± 3 ppt, and dissolved oxygen (DO) of 6–7 mg/L. Each temperature condition was maintained in a single tank to minimize temperature fluctuations and ensure consistent thermal conditions throughout the experimental period; therefore, all fish within a given temperature condition, including the six fish sampled at each time point, were obtained from this single corresponding tank. The fish were acclimated to the experimental conditions for 2 weeks prior to the experiment and fed a commercial diet for black rockfish (Jeil Feed, Daejeon, Republic of Korea) once daily at a rate of 0.5% of their body weight. To minimize the potential effects of feeding on the pharmacokinetic parameters, feeding was discontinued 2 days before drug administration and throughout the experimental period. No mortality was observed during the experimental period. All experimental procedures involving animals were conducted in accordance with the ethical guidelines approved by the Institutional Animal Care and Use Committee of Kunsan National University, Republic of Korea.

### 2.3. Enrofloxacin Administration and Sampling

ENR was administered intraperitoneally to Black rockfish at a single dose of 10 mg/kg body weight using a 1 mL disposable syringe. The ENR dosing solution was prepared such that each fish received an injection volume of 0.1 mL to achieve the target dose of 10 mg/kg body weight, using a 1 mL disposable syringe fitted with a 26-gauge needle. Injections were administered into the peritoneal cavity, taking care to avoid puncturing internal organs. No anesthetic agents were used during drug administration, as the potential effects of anesthesia on fish pharmacokinetics remain unclear and represent a limitation of the field; instead, to minimize handling stress and any potential influence of anesthesia on early absorption and distribution kinetics, fish were gently restrained by covering them with a wet cloth during injection, and all procedures were performed by experienced personnel to ensure rapid and consistent handling. Blood and tissue samples were collected at 0.5, 1, 2, 3, 6, 9, 12, 24, 48, 72, 96, 120, and 168 h after ENR administration, with six fish sampled at each time point. To ensure accurate sample collection at the earliest time points, fish assigned to the 0.5 h time point (six fish) were injected first, with all six fish injected within 3 min and immediately transferred to an isolated, netted enclosure within the same temperature-controlled tank until sampling; blood and tissue samples were then collected and processed at the corresponding time point. Only after this procedure, including sampling and dissection, had been completed for the 0.5 h time point were fish assigned to the 1 h time point injected, following the same procedure. Fish assigned to the remaining time points (2 to 168 h) were injected as a single batch and returned to their original holding tank, from which fish were subsequently sampled at each designated time point. At each sampling time point, fish were anesthetized with MS-222 (approximately 50 ppm), and blood samples were collected from the caudal vein using serum separator tubes (Becton Dickinson, Franklin Lakes, NJ, USA). The blood samples were centrifuged at 4500× *g* for 10 min at 4 °C to obtain serum. Following blood collection, the liver, kidney, and muscle tissues were immediately collected from each fish. Serum and tissue samples were stored at −80 °C until HPLC–MS/MS analysis.

### 2.4. HPLC–MS/MS Analysis of Enrofloxacin and Ciprofloxacin

ENR and CIP in serum and tissue samples were analyzed using a Poroshell 120 EC-C18 column (2.1 mm × 150 mm, 1.8 μm; Agilent Technologies, Santa Clara, CA, USA) installed on a QSight LX50 HPLC system coupled to a QSight 420 triple quadrupole mass spectrometer (PerkinElmer, Waltham, MA, USA), according to the Korean Food Standards [31]. Homogenized serum (0.2 mL), liver (1.0 g), kidney (0.2 g), and muscle (2.0 g) samples were transferred to 50 mL centrifuge tubes and mixed with acetonitrile at a ratio of five times the sample volume or weight, as appropriate. The samples were vortexed for 10 min and then centrifuged at 4800× *g* for 10 min at 4 °C. The resulting supernatants were transferred to new 50 mL centrifuge tubes. C18 sorbent was added at 50 mg per mL of the initial acetonitrile volume, and an equal volume of acetonitrile-saturated n-hexane was then added to the supernatants. The mixtures were vortexed for 5 min and then centrifuged at 4800× *g* for 10 min at 4 °C. After removing the acetonitrile-saturated n-hexane layer, the supernatants were concentrated under a stream of nitrogen at 40 °C and reconstituted in 1 mL of mobile phase. The samples were then filtered through a 0.2 μm PTFE syringe filter prior to analysis.

The mobile phase consisted of 0.1% formic acid in water (A) and 0.1% formic acid in acetonitrile (B), delivered at a flow rate of 0.3 mL/min. The gradient started at 95% mobile phase A, decreased to 40% at 5.0 min and to 0% at 5.5 min, and was maintained at 0% until 9.5 min. The composition was then returned to the initial 95% level over 0.2 min and maintained until 11.5 min. The injection volume was 10 μL, and ENR and CIP were detected in positive electrospray ionization (ESI+) mode. For ENR, the transition from *m*/*z* 360 to 316 was used for quantification, while transitions from *m*/*z* 360 to 342 and 245 were used for qualitative identification. For CIP, the transition from *m*/*z* 332 to 314 was used for quantification, while transitions from *m*/*z* 332 to 245 and 231 were used for qualitative identification.

The analytical method was validated by spiking blank serum and tissue samples with ENR and CIP, and quantification was performed using matrix-matched calibration. Calibration curves for ENR and CIP in serum and tissue matrices were linear over the concentration range of 0.01–0.4 μg/mL, with coefficients of determination (R^2^) greater than 0.9964. Accuracy and precision were expressed as recovery and coefficient of variation, respectively. Serum and tissue samples with ENR or CIP concentrations exceeding the upper limit of the calibration range were diluted with blank matrix extract prior to analysis to bring the concentration within the calibration range. Both parameters were evaluated in serum and all tissue matrices at concentrations of 0.01 and 0.05 μg/mL, with three replicate measurements at each concentration. The recoveries of ENR and CIP across all matrices ranged from 78.3% to 97.5% and from 80.5% to 107.2%, respectively. For ENR, the intra-day and inter-day precision values ranged from 5.5% to 13.5% and from 8.7% to 12.6%, respectively. For CIP, the corresponding values ranged from 2.6% to 14.4% and from 3.5% to 12.8%, respectively. The limits of detection (LOD) and quantification (LOQ) were calculated from the standard deviation of the y-intercept (σ) and the slope (S) of the calibration curves using the following equations: LOD = 3.3 × σ/S and LOQ = 10.0 × σ/S. For ENR, the LOD and LOQ ranged from 0.001 to 0.002 μg/mL (μg/g) and from 0.002 to 0.007 μg/mL (μg/g), respectively. For CIP, the corresponding LOD and LOQ values ranged from 0.001 to 0.002 μg/mL (μg/g) and from 0.001 to 0.006 μg/mL (μg/g), respectively.

### 2.5. Statistical Analysis

Statistical analysis was performed using GraphPad Prism 10 (GraphPad Software, San Diego, CA, USA). Since serum and tissue samples were obtained from different individual fish at each sampling time point, the concentration data were treated as independent, unpaired observations rather than repeated measures. Given this independent sampling design and the likelihood that concentration data would not follow a normal distribution, the Mann–Whitney U test, a non-parametric method for comparing two independent groups, was applied to compare ENR and CIP concentrations between the two water temperatures (13 °C and 22 °C) at each sampling time point. A *p*-value of less than 0.05 was considered statistically significant, and all data are expressed as mean ± standard deviation (SD).

### 2.6. Pharmacokinetic Analysis

Pharmacokinetic analysis was performed using PKSolver software (version 2.0) [32]. Because destructive sampling was used, with six different fish sampled at each time point, individual concentration–time profiles could not be obtained for each fish; therefore, the PK parameters of ENR were estimated using naiv-pooled approach, in which mean concentrations at each time point, obtained from the serum and tissues of six fish, were used to construct a single concentration–time curve for model fitting.

The PK parameters of ENR were estimated using a two-compartment model, whereas those of its metabolite, CIP, were determined using a non-compartmental analysis. The following pharmacokinetic parameters were determined: distribution rate constant (α), elimination rate constant (β), absorption rate constant (K_a_), transport rate constant from central compartment to peripheral compartment (K_12_), transport rate constant from peripheral compartment to central compartment (K_21_), elimination rate constant from the central compartment (K_10_), absorption half-life (t_1/2Ka_), distribution half-life (t_1/2α_), elimination half-life (t_1/2β_), maximum concentration (C_max_), time to peak concentration (T_max_), area under the concentration–time curve (AUC_0-inf_), volume of distribution of the central compartment relative to bioavailability (V/F), clearance relative to bioavailability (CL/F), and mean residence time (MRT). Because intraperitoneal administration is an extravascular route, complete systemic bioavailability (F) could not be assumed; therefore, V/F and CL/F represent apparent parameters relative to bioavailability rather than absolute volume of distribution or clearance, and were interpreted accordingly. The proportion of ENR metabolically converted to CIP was calculated using the following equation [33]: The apparent metabolic rate (AMR) = AUC_CIP_/(AUC_ENR_ + AUC_CIP_) × 100.

## 3. Results

Following intraperitoneal administration of ENR at 13 °C and 22 °C, the serum concentration–time data of ENR were best described by a two-compartment model. Because pharmacokinetic parameters throughout this study were estimated from a single concentration–time curve fitted to mean concentration data at each time point, formal statistical comparisons between temperatures were not performed for any of the pharmacokinetic parameters reported below; therefore, numerical differences described in this section reflect observed values rather than statistically confirmed differences. The major pharmacokinetic parameters of ENR obtained from serum at 13 °C and 22 °C are presented in Table 1. K_a_ of ENR was calculated as 4.23 h^−1^ at 13 °C and 31.16 h^−1^ at 22 °C, with corresponding t_1/2Ka_ values of 0.16 h and 0.02 h, respectively. K_12_ differed between water temperatures, with values of 0.58 h^−1^ at 13 °C and 0.37 h^−1^ at 22 °C, whereas K_21_ showed no marked difference between temperatures, with values of 0.19 h^−1^ and 0.18 h^−1^ at 13 °C and 22 °C, respectively. t_1/2α_ was 0.83 h at 13 °C and 1.10 h at 22 °C, while t_1/2β_ was 35.75 h at 13 °C and 21.11 h at 22 °C, indicating a marked effect of temperature. T_max_ and C_max_ were 0.53 h and 12.99 µg/mL at 13 °C, and 0.14 h and 6.76 µg/mL at 22 °C, respectively. CL/F was 0.05 L/h/kg at 13 °C and 0.16 L/h/kg at 22 °C, while MRT was 47.79 h at 13 °C and 26.49 h at 22 °C. V/F was 0.55 L/kg at 13 °C and 1.39 L/kg at 22 °C. AUC_0-inf_ was 214.57 and 62.45·μg/mL at 13 °C and 22 °C, respectively.

CIP, the metabolite of ENR, was detected at relatively low concentrations, and the serum concentration–time data of CIP could not be described by a compartmental model. Therefore, C_max_, T_max_, t_1/2β_, and AUC_0-inf_ of CIP were determined using non-compartmental analysis (Table 2). The serum C_max_ of CIP was 0.84 µg/mL at 22 °C, higher than 0.42 µg/mL at 13 °C. The t_1/2β_ was longer at 13 °C (20.24 h) than at 22 °C (8.18 h), while the AUC_0-inf_ was 4.58 and 5.03 h·μg/mL at 13 °C and 22 °C, respectively. AMR was 8.05% at 22 °C, approximately 3.8-fold higher than at 13 °C (2.13%).

**Figure 1:**
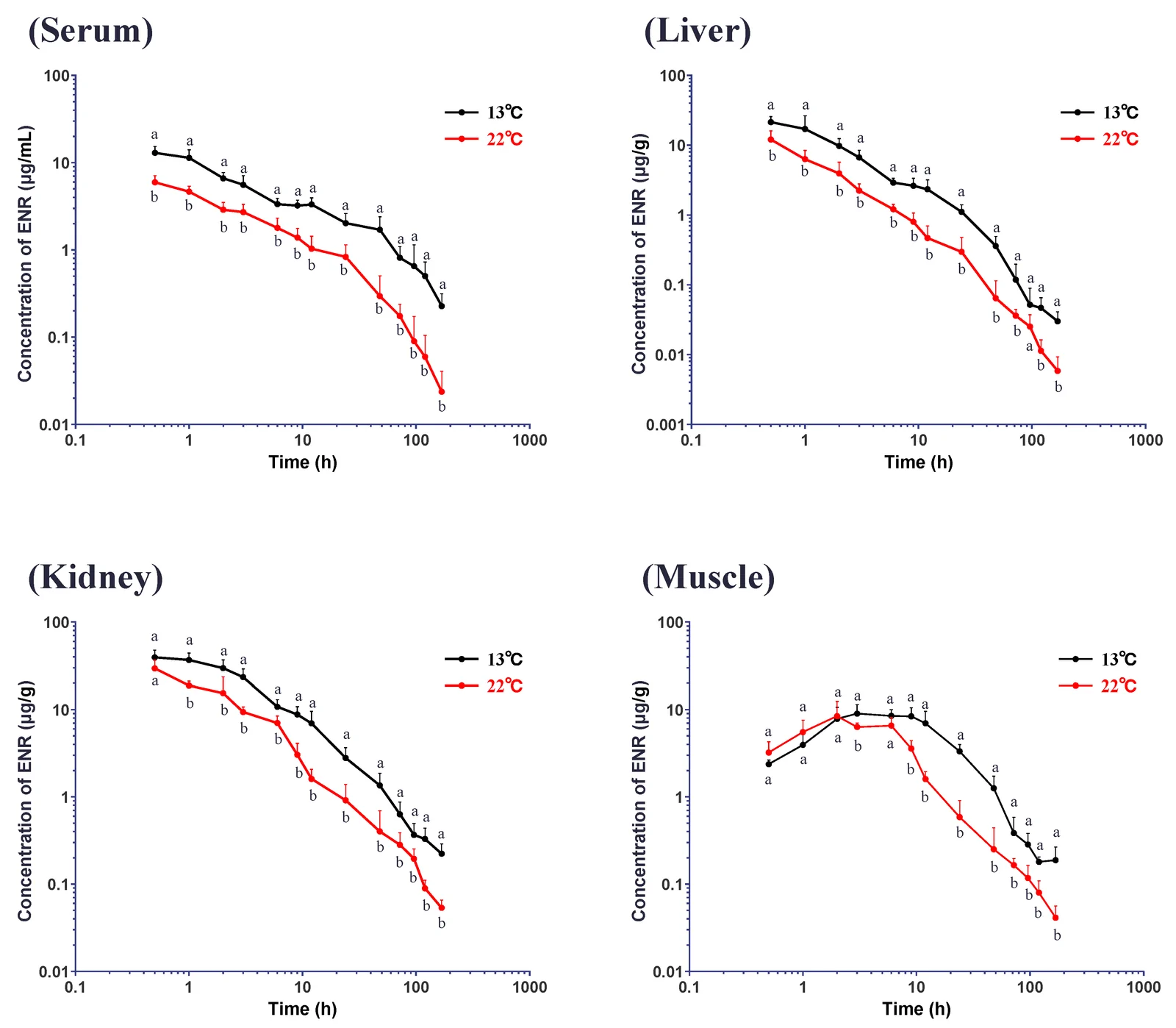
Concentration–time profiles of enrofloxacin (ENR) (mean ± standard deviation, n = 6) in the serum, liver, kidney, and muscle of Black rockfish (*Sebastes schlegelii*) following a single intraperitoneal administration of 10 mg/kg at 13 °C and 22 °C. Data were compared between the two temperature conditions at each sampling time point using the Mann–Whitney test. Statistical significance was set at *p* < 0.05. Different lowercase letters (a, b) above the data points indicate statistically significant differences between temperatures at each time point, while identical letters indicate no significant difference.

Tissue concentrations of ENR obtained after intraperitoneal administration of ENR at 10 mg/kg in Black rockfish are presented in Figure 1 (raw concentration data are provided in Supplementary Table S1). ENR concentrations in serum, liver, and kidney were significantly higher at 13 °C than at 22 °C at most sampling time points (*p* < 0.05), whereas muscle concentrations showed significant temperature-dependent differences mainly during the elimination phase. At 22 °C, ENR concentrations in serum, liver, and kidney reached maximum concentrations at the first sampling time point (0.5 h post-administration), with values of 5.95 ± 1.15 μg/mL, 12.03 ± 4.00 μg/g, and 29.47 ± 7.07 μg/g, respectively. ENR concentrations declined rapidly in serum and liver until 6 h and in kidney until 12 h post-administration, reaching 1.79 ± 0.52 μg/mL, 1.21 ± 0.22 μg/g, and 1.61 ± 0.46 μg/g, respectively. Thereafter, ENR concentrations in all three tissues showed a relatively gradual decline until 168 h. In contrast, the ENR concentration in muscle increased to a maximum of 8.42 ± 3.98 μg/g at 2 h post-administration and remained relatively constant until 6 h. Thereafter, it declined rapidly until 24 h, reaching 0.59 ± 0.32 μg/g, followed by a relatively gradual decline until 168 h. At 13 °C, ENR concentrations were generally higher than those at 22 °C across all tissues, although the concentration–time profiles of each tissue were similar to those observed at 22 °C. Maximum concentrations of ENR in serum, liver, and kidney were observed at 0.5 h post-administration, with values of 12.94 ± 2.37 μg/mL, 21.37 ± 4.40 μg/g, and 39.46 ± 8.15 μg/g, respectively. These concentrations declined rapidly in serum and liver until 6 h and in kidney until 12 h post-administration, reaching 3.34 ± 0.56 μg/mL, 2.90 ± 0.45 μg/g, and 6.97 ± 2.57 μg/g, respectively. Thereafter, ENR concentrations in all three tissues showed a relatively gradual decline until 168 h. In muscle, the ENR concentration reached a maximum of 9.01 ± 2.38 μg/g at 3 h post-administration and remained relatively constant until 9 h. It then declined rapidly to 1.25 ± 0.46 μg/g by 48 h, followed by a gradual decline, reaching 0.19 ± 0.08 μg/g at 168 h.

**Figure 2:**
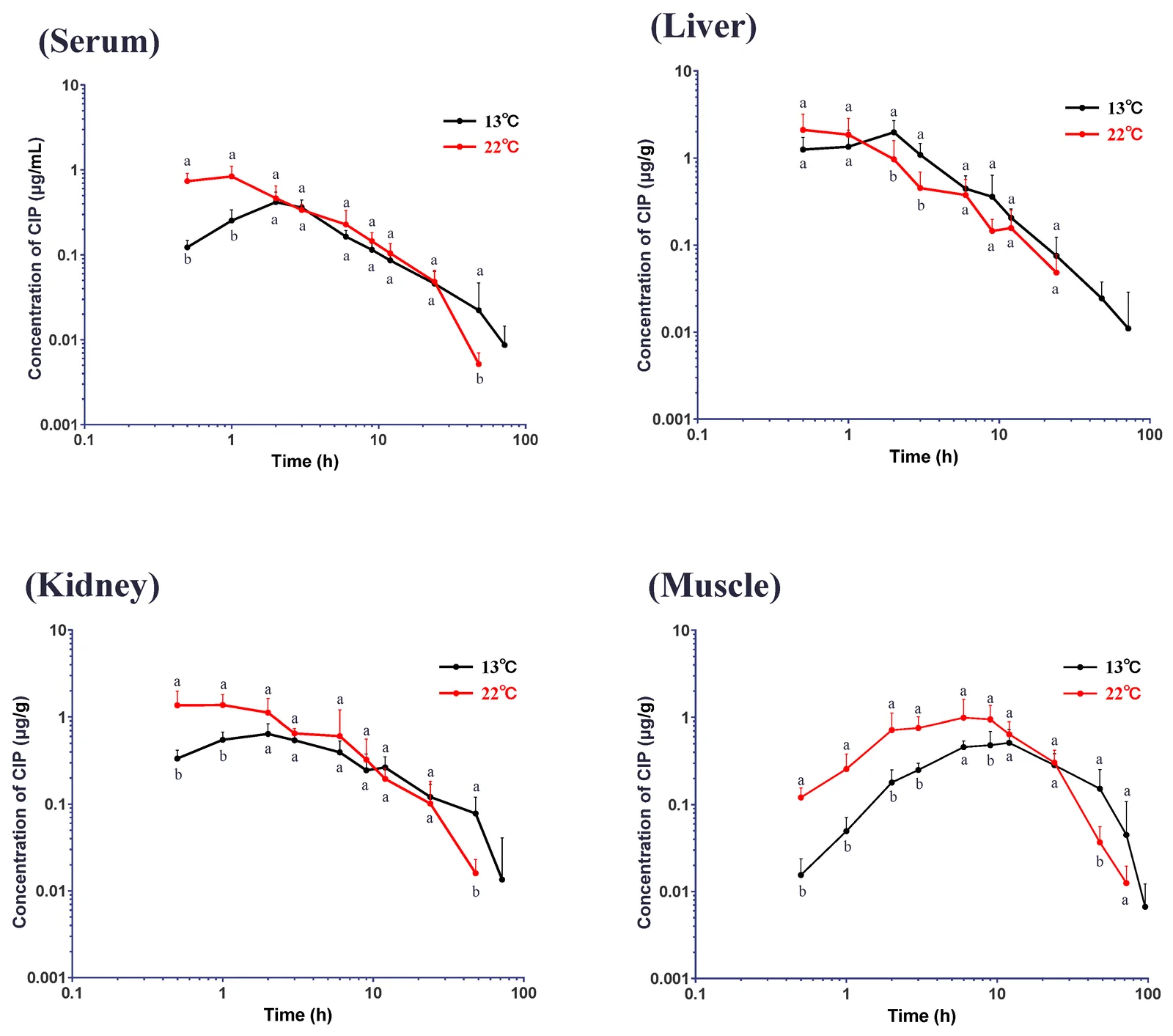
Concentration–time profiles of ciprofloxacin (CIP), the metabolite of enrofloxacin (mean ± standard deviation, n = 6), in the serum, liver, kidney, and muscle of Black rockfish (*Sebastes schlegelii*) following a single intraperitoneal administration of ENR at 10 mg/kg at 13 °C and 22 °C. Data were compared between the two temperature conditions at each sampling time point using the Mann–Whitney test. Statistical significance was set at *p* < 0.05. Different lowercase letters (a, b) above the data points indicate statistically significant differences between temperatures at each time point, while identical letters indicate no significant difference.

Tissue concentrations of CIP obtained after intraperitoneal administration of ENR at 10 mg/kg in Black rockfish are presented in Figure 2 (raw concentration data are provided in Supplementary Table S2). CIP concentrations were significantly higher at 22 °C than at 13 °C during the early phase (*p* < 0.05); however, concentrations at 22 °C subsequently declined more rapidly, becoming comparable to those at 13 °C, and CIP fell below the limit of quantification earlier at 22 °C than at 13 °C in all tissues. CIP was detected in all tissues from 0.5 h post-administration under both temperature conditions. At 22 °C, CIP was detected in serum, liver, kidney, and muscle until 48, 24, 48, and 96 h post-administration, respectively, whereas at 13 °C, CIP was detected in all tissues until 72 h post-administration. The maximum concentrations of CIP in all tissues were higher at 22 °C than at 13 °C. In serum, the maximum CIP concentration was 0.84 ± 0.24 μg/mL at 1 h post-administration at 22 °C, compared with 0.42 ± 0.13 μg/mL at 2 h post-administration at 13 °C. The liver showed the highest CIP concentrations among all tissues. At 22 °C, the CIP concentration in the liver reached a maximum of 2.11 ± 1.08 μg/g at 0.5 h post-administration, whereas at 13 °C, it reached a maximum of 1.97 ± 0.72 μg/g at 2 h post-administration. In the kidney, the maximum CIP concentrations were 0.64 ± 0.20 μg/g and 1.37 ± 0.44 μg/g at 13 °C and 22 °C, respectively, with the maximum concentrations reached at 2 h and 0.5 h post-administration, respectively. CIP reached its maximum concentration in muscle later than in the other tissues. At 13 °C, the maximum concentration was 0.51 ± 0.21 μg/g at 12 h post-administration, whereas at 22 °C, it was 0.99 ± 0.61 μg/g at 6 h post-administration.

## 4. Discussion

In this study, we investigated the serum pharmacokinetics and tissue distribution of ENR and its metabolite CIP in black rockfish following a single intraperitoneal administration of ENR (10 mg/kg) at two water temperatures (13 °C and 22 °C). A limitation of the present study is that fish at each water temperature were maintained in a single 600 L tank rather than in multiple independent tank replicates; therefore, water temperature and tank were completely confounded, and tank-specific environmental effects could not be statistically separated from the effects of water temperature. This design was adopted to minimize temperature fluctuations and ensure consistent thermal conditions throughout the experimental period. Because a naïve-pooled approach based on destructive sampling was used in the present study, the assessment of parameter uncertainty based on inter-individual variability was inherently not possible. Model fitting was performed using uniform weighting (W = 1), and standard errors or 95% confidence intervals for the estimated pharmacokinetic parameters were not reported. In addition, statistical comparisons between the two water temperatures were performed separately at each sampling time point using the Mann–Whitney U test, without correction for multiple comparisons across time points; therefore, the possibility of an inflated type I error rate due to repeated testing should be acknowledged as a limitation of the statistical analysis. In black rockfish, the serum concentration–time data of ENR were best described by a two-compartment model, consistent with findings in brown trout, sea bass, European cuttlefish, and Chinese mitten-handed crab [15,34,35,36]. Model selection was based on goodness-of-fit criteria, including the coefficient of determination (R^2^), Akaike Information Criterion (AIC), and Schwarz Criterion (SC). The two-compartment model showed a markedly better fit than the one-compartment model at both temperatures (13 °C: R^2^, 0.998 vs. 0.945; AIC, 7.08 vs. 46.58; SC, 9.91 vs. 48.27; 22 °C: R^2^, 0.996 vs. 0.964; AIC, −13.35 vs. 19.74; SC, −10.53 vs. 21.43), supporting the selection of the two-compartment model for describing ENR pharmacokinetics in black rockfish. However, because the estimated t_1/2Ka_ at 22 °C was only 0.02 h, the absorption phase may have been almost entirely completed before the first sampling time point (0.5 h), raising concern regarding the reliability of the estimated Ka and t_1/2Ka_ values. In the absence of earlier sampling data, the interpretation of absorption-related differences between temperatures in the present study should therefore be regarded with substantial caution.

Previous studies have reported multiple-peak phenomena, presumably attributable to enterohepatic circulation, for ENR in some fish species, including seabream, silver crucian carp, yellow catfish, crayfish, and yellow river carp [37,38,39,40,41,42]. However, in the present study, no multiple peaks were observed in black rockfish, consistent with findings in sea bass, brown Trout, and rainbow trout [34,43,44].

An increase in water temperature is known to affect the pharmacokinetic properties of drugs in fish, generally accelerating pharmacokinetic processes such as absorption, distribution, and elimination [45,46,47]. Intraperitoneally administered ENR was absorbed very rapidly, and this absorption was affected by the increase in water temperature. The absorption half-life (t_1/2Ka_) was markedly shorter at 22 °C (0.02 h) than at 13 °C (0.16 h), indicating that ENR is rapidly distributed into the systemic circulation following intraperitoneal administration. In contrast, the distribution half-life (t_1/2α_) was 0.83 h at 13 °C and 1.10 h at 22 °C, a pattern opposite to the temperature dependence observed for the absorption rate. This finding may reflect a limitation arising from the early sampling intervals used in this study. The earliest serum sampling time point in this study was 0.5 h post-administration, at which time ENR had already reached its maximum concentration under both temperature conditions (13 °C: 12.94 μg/mL; 22 °C: 5.95 μg/mL). In particular, given the extremely short t_1/2Ka_ at 22 °C (0.02 h), it is likely that most of the absorption and initial distribution processes immediately following administration had already occurred before the 0.5 h time point. Notably, the ENR concentration in muscle reached its maximum earlier at 22 °C than at 13 °C, showing the typical trend of accelerated distribution at higher temperatures. Therefore, the sampling design of this study may not have adequately captured the early distribution kinetics under high-temperature conditions, and future studies should adjust sampling intervals and evaluate ENR concentrations across additional tissues, such as gill, skin, and gastrointestinal tract, to more clearly elucidate the temperature-dependent characteristics of ENR absorption and tissue distribution in the early post-administration period.

In a two-compartment model, the extent of tissue distribution of a drug can be assessed through the ratio of the intercompartmental transfer rate constants (K_12_/K_21_) [48]. Temperature affected the distribution rate constant from the central to the peripheral compartment (K_12_), but had no clear effect on the reverse transfer rate constant from the peripheral to the central compartment (K_21_). K_12_ was approximately 1.5-fold higher at 13 °C (0.58 h^−1^) than at 22 °C (0.37 h^−1^), whereas K_21_ was similar between the two temperatures (0.19 h^−1^ and 0.18 h^−1^, respectively). At both temperatures, K_12_ was higher than K_21_, and the K_12_/K_21_ ratio was higher at 13 °C than at 22 °C. This suggests a stronger tendency for ENR to be retained in tissues at lower temperature, which is consistent with the measured tissue concentration data (Figure 1). Similar trends have been reported for oxytetracycline in channel catfish, florfenicol in Nile tilapia, oxolinic acid in sea bass, and oxytetracycline and difloxacin in crucian carp [46,47,49,50,51,52]. In contrast, the K_12_/K_21_ ratio of ENR shows species-dependent variation. In mud crab, K_21_ was higher than K_12_ at low temperature, whereas K_12_ was higher at high temperature [53]. In large yellow croaker and turbot, K21 was higher than K12 at both temperatures [9,54].

The elimination rate of ENR in black rockfish was affected by water temperature, with faster elimination observed at higher temperatures. Metabolic and excretory rates in fish are known to increase by approximately 10% for every 1 °C rise in water temperature [55], and this temperature-dependent acceleration of drug elimination is a well-documented phenomenon across ectothermic species. Such acceleration may reflect increased hepatic metabolic activity, enhanced renal perfusion and excretion, and an overall rise in metabolic rate associated with higher water temperature; however, direct evidence for these underlying mechanisms, such as temperature-dependent changes in hepatic enzyme activity or renal blood flow, remains limited across fish species in general and has not been reported in black rockfish. As these physiological parameters were not measured in the present study, the mechanisms underlying the observed temperature-dependent elimination of ENR remain to be elucidated, and further studies directly investigating hepatic and renal function under varying water temperature conditions are warranted in this species. Although the extent to which water temperature affects drug elimination rate varies depending on the drug, fish species, temperature range, route of administration, and experimental conditions, an increase in the elimination rate of drugs with rising water temperature has been confirmed in various fish species, including fingerling rainbow trout, largemouth bass, Nile tilapia, turbot, and sea bass [44,45,46,54,56]. In the present study, the elimination half-life (t_1/2β_) was longer at 13 °C (35.75 h) than at 22 °C (21.11 h), and the higher elimination rate constant from the central compartment (K_10_) at 22 °C supports the finding that the drug was eliminated more rapidly at higher water temperature. Consistent with the parenteral administration route used in the present study, these t_1/2β_ values are similar to those reported in snakehead fish (19.82 h), rainbow trout (24.4–30.4 h), turbot (38.79 h), Atlantic salmon (34.2 h), red pacu (28.9 h), and Korean catfish (17.44 h) [13,44,54,57,58,59].

The liver and kidney are known to be the major sites of ENR accumulation in fish; however, the tissue showing the highest concentration has been reported to differ among fish species. In northern snakehead, grass carp, and crucian carp, the liver has been reported to show higher ENR concentrations than the kidney [60,61,62]. In yellow catfish, similar concentrations were reported in the liver and kidney, whereas in turbot, the kidney showed the highest concentration among the tissues examined [40,54]. In black rockfish, the liver and kidney had markedly higher ENR concentrations than serum regardless of water temperature; in particular, the kidney showed the highest Cmax among the tissues examined. Although this finding suggests that the kidney may play an important role in the accumulation and potential excretion of ENR in black rockfish, concentration data alone are insufficient to establish it as the primary route of excretion. Direct evidence from renal, urinary, biliary, or fecal excretion would be required to confirm this, and further studies are warranted to clarify the major excretory pathway of ENR in this species.

CIP is a metabolite produced through the biotransformation of ENR in vivo [63]. In black rockfish, CIP was formed more rapidly and at higher levels at higher water temperature. The C_max_ of CIP was approximately twice as high at 22 °C (0.84 μg/mL) than at 13 °C (0.42 μg/mL), and the time to reach maximum concentration was also shorter. The t_1/2β_ of CIP was markedly shorter at 22 °C (8.18 h) than at 13 °C (20.24 h), whereas the AUC was similar between the two temperatures (4.58 and 5.03 h·μg/mL at 13 °C and 22 °C, respectively). The AMR, representing the proportion of ENR metabolically converted to CIP based on the AUC of CIP relative to the combined AUCs of ENR and CIP, ranged from 2.13% to 8.05% in black rockfish, indicating that only a small proportion of ENR was converted to CIP. The AMR at 22 °C was approximately 3.8-fold higher than that at 13 °C. In black rockfish, the CYP enzymes involved in ENR metabolism, as well as the effect of water temperature on their activity, have not yet been investigated. Because AMR is derived from the AUCs of CIP and ENR, it reflects the net balance between the formation and elimination of CIP rather than enzyme activity itself. Accordingly, the higher AMR observed at 22 °C may reflect not only enhanced metabolic capacity but also the markedly faster elimination of the parent compound ENR at this temperature. As enzyme activity was not directly measured in the present study, the AMR values reported here should be interpreted as an indirect index of overall metabolic conversion rather than a direct measure of enzymatic biotransformation capacity.

This is consistent with previous reports indicating that aquatic animals exhibit markedly lower metabolic conversion rates than terrestrial animals. In aquatic animals, the AMR has been reported as 1.39% in northern snakehead, 1.84–2.04% in turbot, 3.03% in striped catfish, and 5.88% in red pacu, which are markedly lower than the values reported in terrestrial animals, such as 36% in sheep, 51.5% in pig, 47% in buffalo calf, and 59% and 64% in cow and beef steer, respectively [54,58,60,64,65,66,67,68]. In fish, muscle is the primary edible tissue, and thus the persistence of ENR and CIP in this tissue is directly relevant to food safety. The metabolite CIP can also influence residue levels in muscle. In muscle of black rockfish, CIP was formed to a greater extent at higher water temperature but was eliminated more rapidly, remaining detectable only until 72 h, whereas at lower water temperature, CIP was formed to a lesser extent but was eliminated more slowly, remaining detectable until 96 h. These findings indicate that the elimination of both ENR and CIP is slower at lower water temperature, suggesting that a longer withdrawal period may be required at lower water temperatures.

In a previous study, we reported the pharmacokinetic properties of ENR following a single oral administration to black rockfish under the same water temperature conditions as in the present study [31]. Although drug elimination in fish is generally known to increase with rising water temperature, that previous study instead found that the t_1/2β_ of ENR increased at higher water temperature, indicating slower elimination. A similar finding has been reported in yellow catfish [40]. When the pharmacokinetic properties of ENR in yellow catfish were evaluated at 20 °C, 25 °C, and 30 °C, t_1/2β_ decreased from 20 °C to 25 °C and then increased at 30 °C. This phenomenon was attributed to yellow catfish being a temperature-sensitive species, for which 30 °C exceeds the optimal range, resulting in impaired drug-metabolizing capacity. In the present study, however, when ENR was administered intraperitoneally to the same species, black rockfish, the elimination rate instead increased as water temperature rose from 13 °C to 22 °C, following the general trend of the temperature–elimination relationship, in contrast to the findings for oral administration. In other words, opposite results were obtained depending on the route of administration, despite using the same species under the same water temperature conditions, a discrepancy that cannot be fully explained by temperature-related metabolic changes alone. This discrepancy may be attributable to the occurrence of flip-flop kinetics at high water temperature following oral administration. Flip-flop kinetics is a phenomenon commonly observed with extravascular administration, in which, when the absorption rate is slower than the elimination rate, the terminal slope of the concentration–time curve reflects the ongoing absorption process rather than true elimination, resulting in a terminal phase distinct from that observed after intravenous administration [69]. In a study comparing intravenous, intramuscular, and oral administration of folic acid in pigs, intravenous and intramuscular administration showed similar elimination profiles, whereas oral administration exhibited flip-flop kinetics, in which slow absorption caused the elimination rate to appear slower than it actually was [70].

In yellow catfish, ENR was absorbed most rapidly at 30 °C, suggesting that the prolonged half-life observed in that species is attributable not to delayed absorption but to an actual decline in metabolic capacity. In contrast, orally administered ENR in black rockfish showed slower absorption at higher water temperature (Tmax: 2 h at 13 °C vs. 6 h at 22 °C), a pattern opposite to that observed in yellow catfish. It should be noted, however, that because the previous oral administration study evaluated ENR pharmacokinetics using non-compartmental analysis, absorption and elimination rate constants could not be directly calculated or compared between administration routes; therefore, the following interpretation should be regarded as a plausible hypothesis rather than a confirmed mechanism. This discrepancy raises the possibility that flip-flop kinetics, resulting from delayed absorption rather than impaired metabolic capacity, may offer one possible explanation for the prolonged t_1/2β_ observed in black rockfish following oral administration. Confirming this hypothesis would require reliable separation of absorption and elimination rate constants, ideally supported by an intravenous reference study, which remains unavailable for black rockfish. Further studies directly comparing absorption and elimination kinetics between administration routes are needed to confirm this interpretation.

The comparison between the present study and the previous oral administration study raises the possibility that flip-flop kinetics, arising from delayed absorption following oral drug administration, may be influenced by water temperature in fish. Although this interpretation is based on indirect evidence and warrants further direct investigation, it provides a basis for future research aimed at overcoming the limitations of drug administration in aquaculture. Oral administration remains virtually the only practical route for administering most drugs, including ENR, under commercial aquaculture conditions. Therefore, although research on other routes of administration, such as injection, is also important, studies aimed at improving the absorption and distribution of orally administered drugs are more practically needed. This could be achieved through formulation strategies such as absorption enhancers, lipid-based delivery vehicles, encapsulation, and modified-release formulations. Future studies should investigate such formulation-based approaches together with the temperature dependence of drug absorption, thereby clarifying the relationship between water temperature conditions in aquaculture environments and formulation-based interventions.

## 5. Conclusions

This study evaluated the pharmacokinetic properties and tissue distribution of ENR following intraperitoneal administration to black rockfish at 13 °C and 22 °C. ENR exhibited distinct, temperature-dependent pharmacokinetic properties in black rockfish, with a higher apparent absorption rate and more rapid elimination at higher water temperature, while the greater formation of CIP suggested enhanced overall metabolic conversion at the higher temperature. Regardless of water temperature, the kidney showed the highest ENR concentration among the tissues examined. The metabolite CIP was formed only at very low levels, with greater formation at higher water temperature. Compared with the results of a previous oral administration study conducted under the same conditions, an opposite temperature–elimination pattern was observed following intraperitoneal administration, suggesting that flip-flop kinetics may have occurred at high water temperature following oral administration. These findings suggest that, in evaluating the pharmacokinetic properties of drugs in fish, both water temperature and route of administration should be considered together.

## Figures and Tables

**Table 1:** Pharmacokinetic parameters of enrofloxacin in black rockfish following a single intraperitoneal administration of enrofloxacin at 10 mg/kg body weight at two water temperatures.

Parameters	Unit	13 °C	22 °C
α	h^−1^	0.84	0.63
β	h^−1^	0.02	0.03
K_a_	h^−1^	4.23	31.16
K_12_	h^−1^	0.58	0.37
K_21_	h^−1^	0.19	0.18
K_10_	h^−1^	0.08	0.12
t_1/2α_	h	0.83	1.10
t_1/2β_	h	35.75	21.11
t_1/2Ka_	h	0.16	0.02
CL/F	L/h/kg	0.05	0.16
V/F	L/kg	0.55	1.39
C_max_	µg/mL	12.99	6.76
T_max_	h	0.53	0.14
AUC_0-inf_	h·µg/mL	214.57	62.45
MRT	h	47.79	26.49

Pharmacokinetic parameters were estimated from a single concentration–time curve constructed using mean concentrations from six fish at each sampling time point (n = 6 per time point). α: distribution rate constant; β: elimination rate constant; K_a_: absorption rate constant; K_12_: transport rate constant from central compartment to peripheral compartment; K_21_: transport rate constant from peripheral compartment to central compartment; K_10_: elimination rate constant from the central compartment; t_1/2Ka_: absorption half-life; t_1/2α_: distribution half-life; t_1/2β_: elimination half-life; C_max_: maximum concentration; T_max_: time to peak concentration; AUC_0-inf_: area under the concentration–time curve; V/F: volume of distribution of the central compartment relative to bioavailability; CL/F: clearance relative to bioavailability; MRT: mean residence time.

**Table 2:** Pharmacokinetic parameters of ciprofloxacin in black rockfish following a single intraperitoneal administration of enrofloxacin at 10 mg/kg body weight at two water temperatures.

Parameters	Unit	13 °C	22 °C
C_max_	µg/mL	0.42	0.84
T_max_	h	2	1
t_1/2β_	h	20.24	8.18
AUC_0-inf_	h·µg/mL	4.58	5.03
AMR	%	2.13	8.05

Pharmacokinetic parameters were estimated from a single concentration–time curve constructed using mean concentrations from six fish at each sampling time point (n = 6 per time point). C_max_: maximum concentration; T_max_: time to peak concentration; t_1/2β_: elimination half-life; AUC_0-inf_: area under the concentration–time curve; AMR: apparent metabolic rate.

## Data Availability

The original contributions presented in this study are included in the article. Further inquiries can be directed to the corresponding authors.

## Supplementary Materials

The following supporting information can be downloaded at https://www.mdpi.com/article/10.3390/ani16193034/s1. Table S1: Concentrations of enrofloxacin (ENR) in serum and tissues of black rockfish at two water temperatures; Table S2: Concentrations of ciprofloxacin (CIP) in serum and tissues of black rockfish at two water temperatures.
