# Supplementary material for: Pharmacokinetics and Tissue Distribution of Enrofloxacin and Its Metabolite Ciprofloxacin After a Single Intraperitoneal Administration in Black Rockfish (Sebastes schlegelii) at Two Water Temperatures
Source: Animals (Basel). 2026 Sep 26;16(19):3034. doi: 10.3390/ani16193034 (PMC13663454; doi:10.3390/ani16193034)
Supplement: Supplementary file 1 [file animals-16-03034-s001.zip › animals-4561347-supplementary.pdf]

Table S1. ENR concentration in serum (μg/mL), liver (μg/g), kidney (μg/g), and muscle (μg/g) of black rockfish after a single intraperitoneal administration of 10 mg enrofloxacin/kg body weight at two water temperatures (n = 6).

Time (h)	13°C				22°C			
	Serum	Liver	Kidney	Muscle	Serum	Liver	Kidney	Muscle
0.5	12.94±2.37	21.37±4.40	39.46±8.15	2.23±0.27	5.95±1.15	12.03±4.00	29.47±7.07	3.22±1.06
1	11.35±2.71	17.04±9.31	36.75±7.47	3.94±1.59	4.65±0.71	6.30±2.12	18.79±2.35	5.53±2.00
2	6.61±1.11	9.74±2.71	29.63±7.13	7.84±2.76	2.89±0.61	3.94±1.76	15.42±8.13	8.42±3.98
3	5.57±1.50	6.65±1.83	23.41±5.63	9.01±2.38	2.71±0.62	2.24±0.55	9.38±1.34	6.32±0.72
6	3.34±0.56	2.90±0.45	10.78±2.18	8.47±1.551	1.79±0.52	1.21±0.22	7.03±1.39	6.56±1.52
9	3.22±0.49	2.63±0.74	8.82±1.95	8.38±2.11	1.38±0.38	0.80±0.27	3.04±1.07	3.57±0.84
12	3.32±0.64	2.33±0.86	6.97±2.57	6.97±2.56	1.03±0.40	0.47±0.23	1.61±0.46	1.60±0.34
24	2.03±0.59	1.10±0.29	2.80±0.88	3.33±0.65	0.83±0.32	0.30±0.18	0.92±0.47	0.59±0.32
48	1.70±0.69	0.36±0.13	1.36±0.51	1.25±0.46	0.30±0.21	0.06±0.05	0.40±0.29	0.25±0.19
72	0.81±0.28	0.12±0.08	0.63±0.25	0.39±0.20	0.17±0.06	0.04±0.01	0.28±0.11	0.17±0.03
96	0.81±0.28	0.05±0.04	0.37±0.13	0.28±0.10	0.09±0.08	0.02±0.01	0.20±0.06	0.12±0.05
120	0.50±0.23	0.05±0.02	0.33±0.11	0.18±0.03	0.06±0.05	0.01±0.005	0.09±0.02	0.08±0.03
168	0.23±0.09	0.03±0.01	0.22±0.07	0.19±0.08	0.02±0.02	0.01±0.003	0.05±0.01	0.04±0.01

Data are presented as mean ± SD (standard deviation).

Table S2. CIP concentration in serum (μg/mL), liver (μg/g), kidney (μg/g), and muscle (μg/g) of black rockfish after a single intraperitoneal administration of 10 mg enrofloxacin/kg body weight at two water temperatures (n = 6).

Time (h)	13°C				22°C			
	Serum	Liver	Kidney	Muscle	Serum	Liver	Kidney	Muscle
0.5	0.12±0.03	1.25±0.48	0.33±0.08	0.02±0.01	0.74±0.17	2.11±1.08	1.36±0.63	0.12±0.03
1	0.25±0.08	1.34±0.75	0.55±0.13	0.05±0.02	0.84±0.26	1.85±1.01	1.37±0.44	0.26±0.12
2	0.42±0.13	1.97±0.72	0.64±0.20	0.18±0.07	0.46±0.18	0.97±0.62	1.12±0.52	0.72±0.41
3	0.36±0.08	1.09±0.38	0.54±0.10	0.25±0.05	0.34±0.05	0.45±0.24	0.65±0.09	0.76±0.25
6	0.16±0.03	0.44±0.18	0.39±0.14	0.46±0.08	0.23±0.11	0.38±0.19	0.61±0.60	0.99±0.61
9	0.11±0.04	0.36±0.27	0.24±0.13	0.48±0.21	0.14±0.04	0.15±0.05	0.32±0.24	0.95±0.42
12	0.09±0.01	0.21±0.05	0.26±0.09	0.51±0.21	0.10±0.03	0.15±0.10	0.20±0.06	0.64±0.24
24	0.05±0.02	0.08±0.05	0.12±0.05	0.28±0.10	0.05±0.02	0.05±0.03	0.10±0.08	0.30±0.12
48	0.02±0.02	0.02±0.01	0.08±0.04	0.15±0.10	0.01±0.002	N.D.	0.01±0.01	0.04±0.02
72	0.01±0.01	0.01±0.02	0.01±0.03	0.04±0.06	N.D.	N.D.	N.D.	0.01±0.01
96	N.D.	N.D.	N.D.	0.01±0.01	N.D.	N.D.	N.D.	N.D.
120	N.D.	N.D.	N.D.	N.D.	N.D.	N.D.	N.D.	N.D.
168	N.D.	N.D.	N.D.	N.D.	N.D.	N.D.	N.D.	N.D.

Data are presented as mean ± SD (standard deviation). N.D.: not detected.
